# Increased BAT Thermogenesis in Male Mouse Apolipoprotein A4 Transgenic Mice

**DOI:** 10.3390/ijms24044231

**Published:** 2023-02-20

**Authors:** Zachary LaRussa, Hsuan-Chih N. Kuo, Kathryn West, Zhijun Shen, Kevin Wisniewski, Patrick Tso, Karen T. Coschigano, Chunmin C. Lo

**Affiliations:** 1 Department of Biomedical Sciences, Heritage College of Osteopathic Medicine, and Diabetes Institute, Ohio University, Athens, OH 45701, USA; 2 Department of Pathology and Laboratory Medicine, University of Cincinnati, Cincinnati, OH 45237, USA

**Keywords:** thermogenesis, plasma lipids, brown adipose tissue, caloric intake, high-fat diet, obesity

## Abstract

Dietary lipids induce apolipoprotein A4 (APOA4) production and brown adipose tissue (BAT) thermogenesis. Administration of exogenous APOA4 elevates BAT thermogenesis in chow-fed mice, but not high-fat diet (HFD)-fed mice. Chronic feeding of HFD attenuates plasma APOA4 production and BAT thermogenesis in wildtype (WT) mice. In light of these observations, we sought to determine whether steady production of APOA4 could keep BAT thermogenesis elevated, even in the presence of HFD consumption, with an aim toward eventual reduction of body weight, fat mass and plasma lipid levels. Transgenic mice with overexpression of mouse APOA4 in the small intestine (APOA4-Tg mice) produce greater plasma APOA4 than their WT controls, even when fed an atherogenic diet. Thus, we used these mice to investigate the correlation of levels of APOA4 and BAT thermogenesis during HFD consumption. The hypothesis of this study was that overexpression of mouse APOA4 in the small intestine and increased plasma APOA4 production would increase BAT thermogenesis and consequently reduce fat mass and plasma lipids of HFD-fed obese mice. To test this hypothesis, BAT thermogenic proteins, body weight, fat mass, caloric intake, and plasma lipids in male APOA4-Tg mice and WT mice fed either a chow diet or a HFD were measured. When fed a chow diet, APOA4 levels were elevated, plasma triglyceride (TG) levels were reduced, and BAT levels of UCP1 trended upward, while body weight, fat mass, caloric intake, and plasma lipids were comparable between APOA4-Tg and WT mice. After a four-week feeding of HFD, APOA4-Tg mice maintained elevated plasma APOA4 and reduced plasma TG, but UCP1 levels in BAT were significantly elevated in comparison to WT controls; body weight, fat mass and caloric intake were still comparable. After 10-week consumption of HFD, however, while APOA4-Tg mice still exhibited increased plasma APOA4, UCP1 levels and reduced TG levels, a reduction in body weight, fat mass and levels of plasma lipids and leptin were finally observed in comparison to their WT controls and independent of caloric intake. Additionally, APOA4-Tg mice exhibited increased energy expenditure at several time points when measured during the 10-week HFD feeding. Thus, overexpression of APOA4 in the small intestine and maintenance of elevated levels of plasma APOA4 appear to correlate with elevation of UCP1-dependent BAT thermogenesis and subsequent protection against HFD-induced obesity in mice.

## 1. Introduction

Obesity has become a global epidemic, affecting more than 40% of adults (nearly 100 million) in the US [1]. It increases the incidence of type 2 diabetes, cardiovascular diseases, stroke, and metabolic disorders [2,3]. It has been found that obese humans and animals have reduced brown adipose tissue (BAT) thermogenesis [4,5,6]. In contrast, activation of BAT thermogenesis reduces body weight, reduces plasma triglycerides (TG), and increases insulin sensitivity in humans and rodents [7,8,9,10,11]. Thus, searching for a potent stimulant of BAT thermogenesis and elucidating its mechanism of action would provide the best avenue of research for developing new strategies to counter obesity and obesity-related complications [9,12].

Acute feeding of dietary lipids activates BAT thermogenesis in animals and humans to counteract the energy surplus [10,13,14,15]. BAT is intensely innervated by sympathetic nerves [16]. Norepinephrine is released at nerve terminals when sympathetic neurons are stimulated [17,18]. The amount of tyrosine hydroxylase (TH) is the rate-limiting step for the synthesis of norepinephrine [17,18]. Adenosine monophosphate-activated protein kinase (AMPK) is a major cellular energy sensor, and AMPKα1 specifically is involved in modulation of thermogenesis in the adipose tissues [19,20]. Sympathetic stimulation induces activation of the AMPK pathway that leads to an increase in adipose triglyceride lipase (ATGL) and hormone-sensitive lipase (HSL) for intracellular TG hydrolysis in BAT [21,22,23]. Free fatty acids released from intracellular lipolysis enter mitochondria for fatty acid beta oxidation, where UCP1 and UCP3 are involved in generation of heat production [15,24].

Apolipoprotein A4 (APOA4), a 46 kDa lipid-binding glycoprotein, is one of the most abundant proteins produced by the small intestine in the presence of dietary lipids [25]. APOA4 is secreted into the circulation through the lymphatic system [26], and the small intestine contributes approximately 60% of the plasma APOA4 pool [25]. APOA4 produces short-term satiating effects that reduce meal size but increase meal frequency without altering daily caloric intakes [27]. APOA4 also plays important roles in modulating BAT thermogenesis, lipid transport, cholesterol efflux, inflammation, thrombosis, and processes that protect against developing atherosclerosis and obesity [25,28,29,30,31]. Mice with global knockout of APOA4 (APOA4-KO mice) have reduced levels of TH, ATGL, and UCP1-dependent BAT thermogenesis in response to dietary lipids [32]. In contrast, acute administration of APOA4 protein elevates thermogenesis and fatty acid uptake in the BAT of chow-fed WT mice [31,32]. The findings suggest that peripheral effects of APOA4 may stimulate UCP1-dependent BAT thermogenesis and regulate lipid metabolism. Chronic consumption of a high-fat diet (HFD) promotes excess energy intake and fails to induce BAT thermogenesis, leading to excess energy stored as fat and subsequent development of obesity [4,5,6,33]. Previous reports have demonstrated that APOA4 production in the small intestine cannot be induced by dietary lipids when rodents receive an HFD for longer than 2 weeks [34,35,36]. Further, after 3-week feedings of HFD, when mice receive an acute administration of either APOA4 or saline, thermogenesis and fatty acid uptake in the BAT of APOA4-treated mice are comparable with the saline-treated mice [31]. The findings suggest that chronic consumption of HFD attenuates a rise in lipid-induced APOA4 production and prevents APOA4-induced BAT thermogenesis. Whether increasing the levels of endogenous APOA4 can elevate BAT thermogenesis and attenuate body weight gain and plasma lipids in HFD-fed obese mice remains unknown.

Overexpression of mouse or human APOA4 in the mouse small intestine (endogenous expression) regulates lipid metabolism [29,37]. The transgenic mice with overexpression of mouse APOA4 in the small intestine (APOA4-Tg mice) have multiple tandem copies of a 7 kb DNA fragment containing the entire APOA4 gene and several kb of flanking DNA, including regulatory sequences leading to increased expression of ApoA4 mRNA in the intestine but no other tissue examined [29]. When maintained on a chow diet, APOA4-Tg mice have increased levels of plasma APOA4 protein and comparable body weight and food consumption relative to their WT controls [29]. Additionally, feeding an atherogenic diet consisting of 15% cocoa butter fat, 1.25% cholesterol and 0.5% sodium cholate for 5 days results in even greater intestinal APOA4 mRNA and protein over basal levels in APOA4-Tg mice in comparison to WT controls. Thus, the transgene appears to contain regulatory signals at least somewhat responsive to dietary fat or some other component of the atherogenic diet. The induction, or suppression, of endogenous APOA4 in the small intestine of APOA4-Tg mice by long-term feeding of an HFD, which differs from an atherogenic diet in that it consists of 60% lard fat, 0.3% cholesterol, but no addition of sodium cholate, remains unknown. The objectives of the present experiments sought to investigate if maintenance of increased levels of endogenous APOA4 in APOA4-Tg mice could enhance BAT thermogenesis after short-term intake of HFD for 4 weeks or long-term feeding of HFD for 10 weeks, and lead to attenuated body weight gain and plasma lipids and thus protection against HFD-induced obesity. The hypothesis of this study is that overexpression of mouse APOA4 in the small intestine would increase BAT thermogenesis and consequently reduce body weight and plasma lipids in HFD-fed mice. In the present studies, body weight gain, feeding behavior, BAT thermogenic and lipolytic proteins, and plasma parameters in APOA4-Tg and WT mice in response to a standard chow diet or 4-week or 10-week consumption of HFD were investigated.

## 2. Results

### 2.1. Body Weight, Fat Mass, Plasma Parameters and BAT Thermogenic and Lipolytic Proteins in APOA4-Tg Mice Fed a Chow Diet

**Figure 1:**
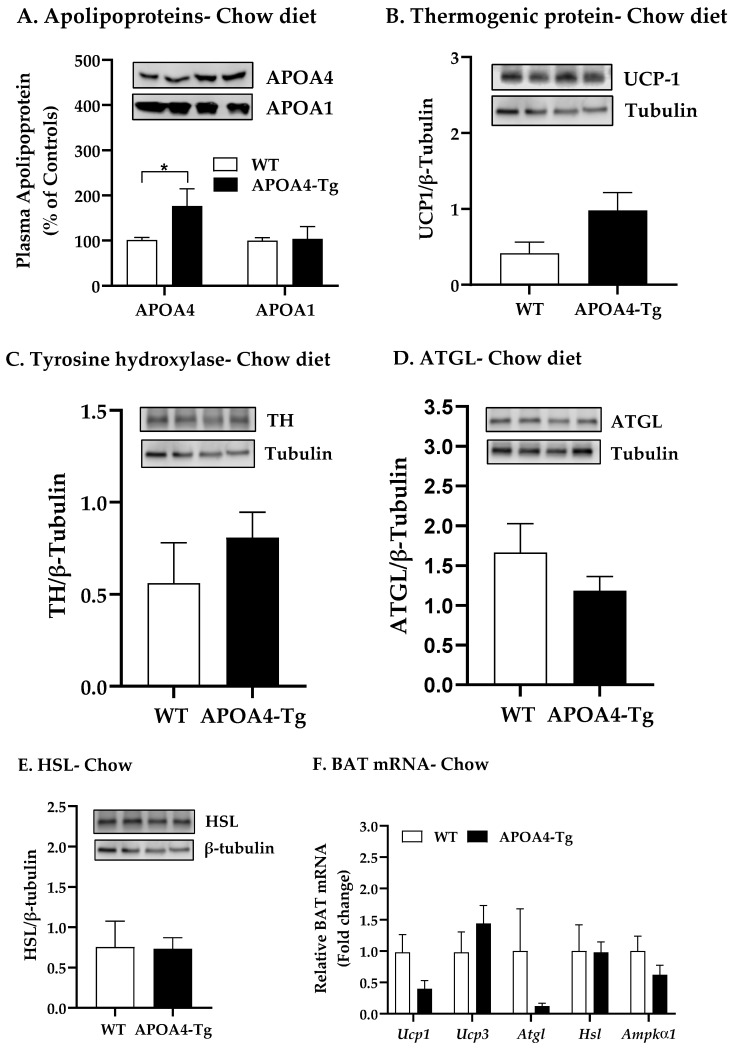
Levels of plasma apolipoproteins (**A**), thermogenic protein (**B**), tyrosine hydroxylase (TH) protein (**C**), adipose triglyceride lipase (ATGL) protein (**D**), hormone sensitive lipase (HSL) protein (**E**), and mRNA (**F**) in BAT of WT and APOA4-Tg mice when maintained on a chow diet at 21 °C. Plasma and BAT in 5 h fasted mice were collected. Plasma apolipoproteins in WT mice were considered as 100%. Data are expressed as mean ± SEM for 6–8 animals per group. Values with asterisks (*) represent significant differences relative to the WT mice (*p* < 0.05).

Acute intraperitoneal administration of APOA4 induces sympathetic activity and stimulates BAT thermogenesis in chow-diet-fed mice at normal ambient temperature [31]. To characterize the effect of overexpression of endogenous APOA4 in a control of BAT thermogenesis at normal ambient temperature, body weight and food intake of WT and APOA4-Tg mice fed a chow diet when they were maintained at 21 °C were monitored. After a 5 h fast, plasma APOA4 protein was nearly two-fold higher in APOA4-Tg mice in comparison to WT mice (*p* < 0.05, Figure 1A). The APOA4-Tg mice produced comparable levels of plasma APOA1 protein relative to WT mice (Figure 1A). Body weights, caloric intake, total fat and lean mass, and fat mass of interscapular BAT, epididymal white adipose tissue (EWAT), and inguinal white adipose tissue (IWAT) were comparable between the two groups (Table 1).

To examine whether the induction of norepinephrine synthesis by overexpression of APOA4 leads to activated AMPK pathway and enhanced intracellular lipolysis and BAT thermogenesis, levels of UCP1, TH, and AMPK pathway and lipolytic enzymes in BAT were determined. Protein levels of UCP1, TH, ATGL, and HSL in BAT were not significantly different between APOA4-Tg mice and their WT controls when maintained on a chow diet at 21 °C (Figure 1B–E). Similarly, no significant differences in levels of *Ucp1*, *Ucp3*, *Atgl*, *Hsl*, and *Ampkα1* mRNA in the BAT were observed between APOA4-Tg and WT mice (Figure 1F).

**Figure 2:**
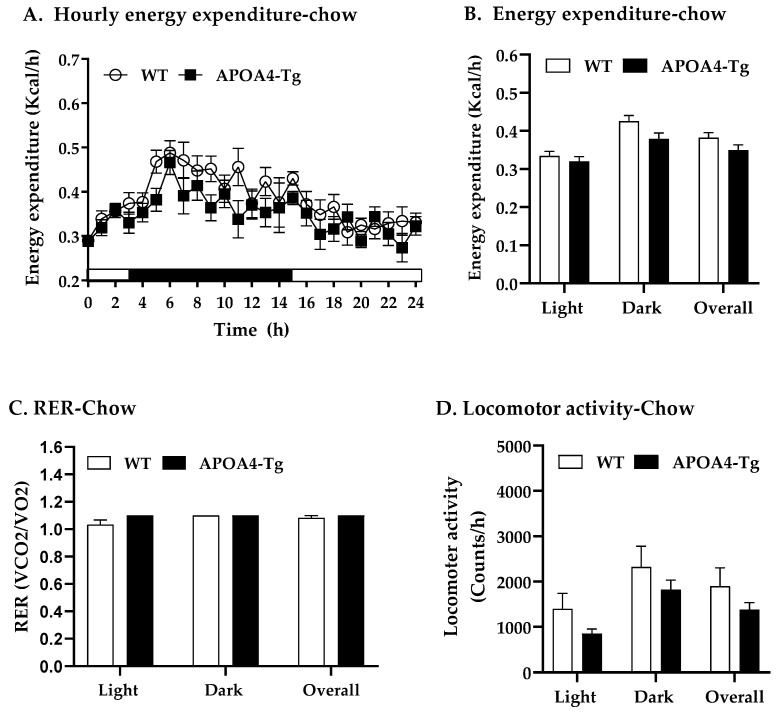
Hourly energy expenditure (**A**), average energy expenditure (**B**), average respiratory exchange ratio (RER, VCO2/VO2) (**C**), and locomotor activity (**D**) during light and dark phases in mice fed a chow diet at 21 °C. Energy expenditure was covaried for lean body mass. Data are expressed as mean ± SEM. *n* = 8 per group.

To test if overexpression of APOA4 alters energy expenditure and locomotor activity, energy expenditure, respiratory exchange ratio (RER), and locomotor activity in chow-fed APOA4-Tg mice were measured. When fed a chow diet, APOA4-Tg mice had comparable lean body mass (22.8 ± 1.3 g) not normalized by body weight relative to WT mice (21.5 ± 0.9 g). No significant differences in hourly EE, average of EE in light or dark phases or overall, RER, and locomotor activity were observed between APOA4-Tg and WT mice when fed a chow diet (Figure 2A–D). APOA4-Tg mice did have reduced levels of plasma TG compared to WT mice (*p* < 0.05; Table 1); levels of plasma cholesterol and leptin were comparable between APOA4-Tg and WT mice (Table 1). Thus, APOA4-Tg mice have elevated plasma APOA4, reduced plasma TG, and comparable levels of UCP1-dependent BAT thermogenesis (as indicated by BAT UCP1 levels), energy expenditure, body weight, caloric intake, fat mass, plasma cholesterol, and plasma leptin when they are maintained on a chow diet at 21 °C.

### 2.2. Body Weight, Fat Mass, Plasma Parameters, and BAT Thermogenic and Lipolytic Proteins in APOA4-Tg Mice Fed an HFD for 4 Weeks

**Figure 3:**
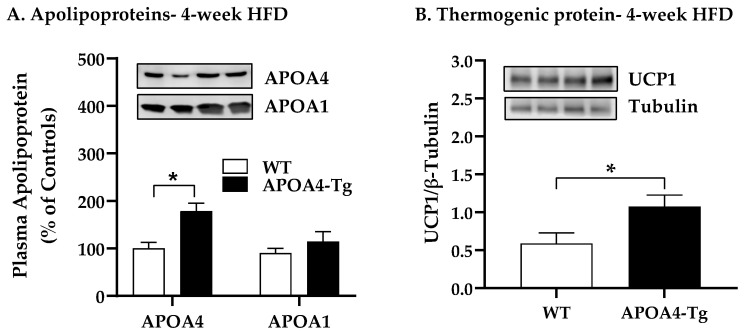

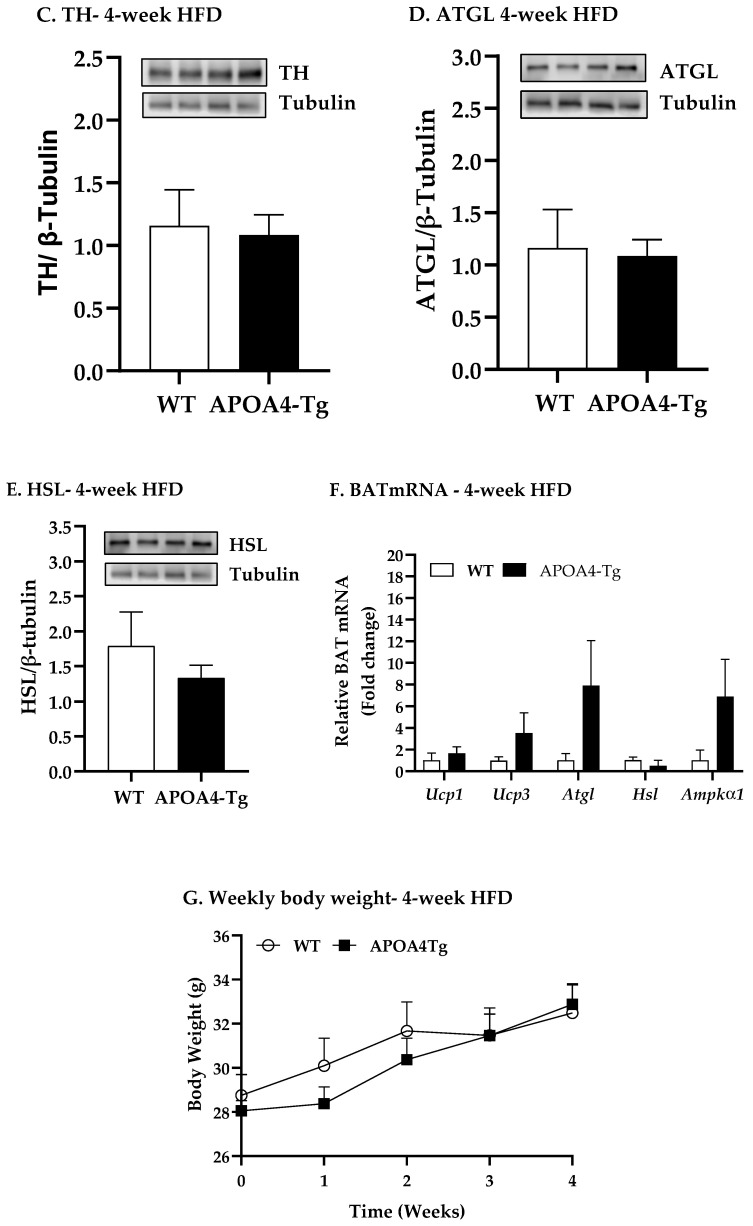
Levels of plasma apolipoproteins (**A**), thermogenic protein (**B**), tyrosine hydroxylase (TH) protein (**C**), adipose triglycerides lipase (ATGL) protein (**D**), hormone-sensitive lipase (HSL) protein (**E**) and mRNA (**F**) in BAT of mice fed HFD for 4 weeks, and weekly body weight (**G**) in WT and APOA4-Tg mice during a 4-week feeding of HFD at 28 °C. Plasma and BAT were collected after a 5 h fast. Plasma APOA4 or APOA1 in WT mice was considered as 100%. Data are expressed as mean ± SEM for 6–8 animals per group. Values with asterisks (*) represent significant differences relative to the WT mice (*p* < 0.05).

To investigate whether increased levels of endogenous APOA4 can modulate BAT thermogenesis and body weight after short-term consumption of an HFD, body weight, fat mass, caloric intake, BAT thermogenic and lipolytic proteins, and plasma lipids in WT and APOA4-Tg mice were measured when they were fed an HFD for 4 weeks. To minimize temperature-induced BAT thermogenesis, APOA4-Tg and WT mice were maintained at a thermoneutral housing temperature (28 ± 0.5 °C, minimal BAT activity) [15]. Our preliminary data showed that this increased temperature had no effect on body weight, food intake, and plasma lipids in APOA4-Tg mice in comparison with WT mice when mice were fed a chow diet at 28 °C. After 4 weeks of HFD, APOA4-Tg mice had higher levels of plasma APOA4 than WT mice (*p* < 0.05; Figure 3A). In contrast, plasma APOA1 protein levels were similar between the two genotypes (Figure 3A). APOA4-Tg mice had significantly higher levels of BAT UCP1 than WT mice (*p* < 0.05; Figure 3B). No significant differences in protein levels of TH, ATGL, and HSL in BAT, and BAT mRNA of *Ucp1*, *Ucp3*, *Atgl*, *Hsl*, and *Ampkα1* were observed between APOA4-Tg and WT mice after 4 weeks of an HFD (Figure 3C–E). During the 4 weeks of HFD feeding, the average weekly body weights of the APOA4-Tg and WT mice were comparable (Figure 3G). After 4 weeks of HFD feeding, APOA4-Tg mice exhibited comparable body weights, average daily caloric intake, total fat and lean mass, and fat mass of interscapular BAT, EWAT, and IWAT relative to the WT mice (Table 2). In addition, APOA4-Tg mice had reduced levels of plasma TG (*p* < 0.05, Table 2) and comparable levels of plasma cholesterol and leptin in response to 4 weeks of HFD (Table 2). The findings suggest that plasma APOA4 levels in APOA4-Tg mice over-expressing APOA4 are not suppressed by short-term feeding of an HFD, and they elevate UCP1-dependent BAT thermogenesis and reduce plasma TG as compared to WT mice when housed under thermoneutral conditions, but do not alter body weight, caloric intake, and levels of plasma cholesterol and leptin.

### 2.3. Body Weight, Fat Mass, Plasma Parameters, and BAT Thermogenic and Lipolytic Proteins in APOA4-Tg Mice Fed an HFD for 10 Weeks

**Figure 4:**
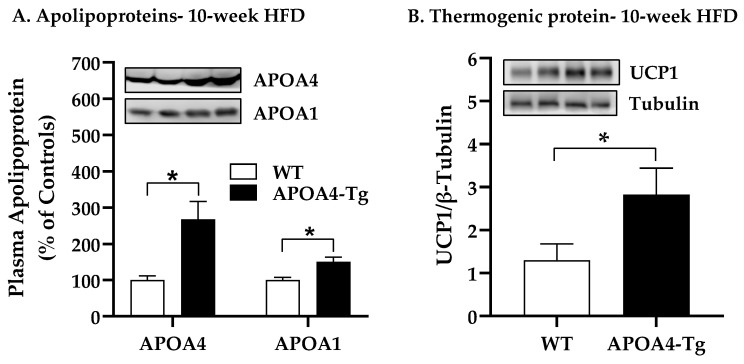

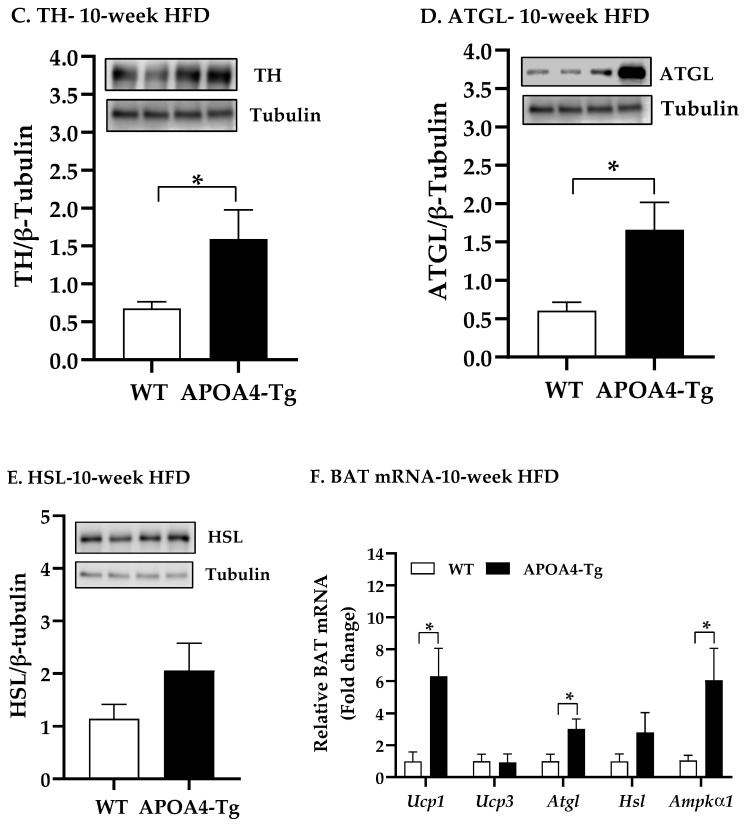
Levels of plasma apolipoproteins (**A**), thermogenic protein (**B**), tyrosine hydroxylase (TH) protein (**C**), adipose triglycerides lipase (ATGL) protein (**D**), hormone-sensitive lipase (HSL) protein (**E**) and mRNA (**F**) in BAT of APOA4-Tg and WT mice. Mice received a 10-week feeding of HFD while housed at 28 °C. After a 5 h fast, plasma and BAT of APOA4-Tg and WT mice were collected. Plasma APOA4 or APOA1 in WT mice was considered as 100%. Data are expressed as mean ± SEM for 6–7 animals per group. Values with asterisks (*) represent significant differences relative to the WT mice (*p* < 0.05).

To investigate whether endogenous APOA4 production in APOA4-Tg mice can be induced by chronic consumption of an HFD, and whether increased levels of endogenous APOA4 can modulate BAT thermogenesis and lipid metabolism, BAT thermogenic and lipolytic proteins, body weight, caloric intake, and plasma lipids in WT and APOA4-Tg mice were measured when they were fed an HFD for 10 weeks at 28 °C. APOA4-Tg mice produced nearly three-fold more plasma APOA4 and two-fold more APOA1 compared with WT mice (*p* < 0.05; Figure 4A). Levels of UCP1, TH, and ATGL proteins in the BAT of APOA4-Tg mice were significantly greater than those in WT mice (*p* < 0.05, Figure 4B–D). APOA4-Tg mice had a tendency toward increased BAT HSL protein levels relative to WT mice (Figure 4E). Relative to WT mice, APOA4-Tg mice had upregulated levels of *Ucp1*, *Atgl*, and *Ampkα1* gene expression in BAT (*p*< 0.05, Figure 4F). No significant differences in *Ucp3* and *Hsl* gene expression levels in BAT were observed between APOA4-Tg and WT mice (Figure 4F).

**Figure 5:**
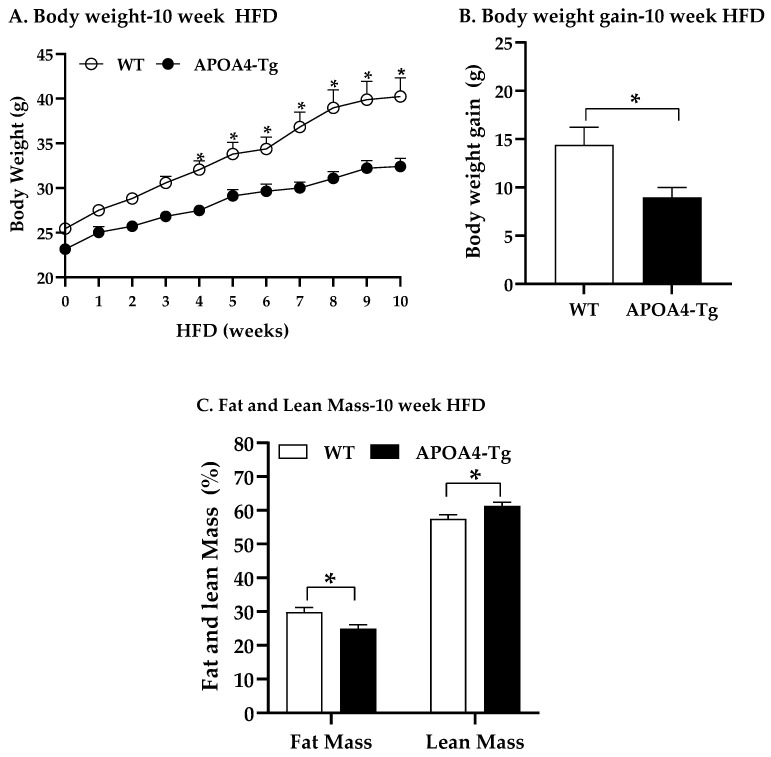
Body weight (**A**) in mice during a 10-week feeding of HFD, and body weight gain (**B**) and percent fat and lean mass (**C**) in mice after 10 weeks of HFD at 28 °C. Data are expressed as mean ± SEM for nine animals per group. Values with asterisks (*) represent significant differences relative to the WT mice (*p* < 0.05).

Body weights of WT and APOA4-Tg mice increased throughout the 10-week feeding (Figure 5A). During the first 3 weeks of the HFD, weekly body weights of APOA4-Tg mice were comparable to WT mice (Figure 5A). In contrast, body weights of APOA4-Tg mice were significantly less than WT mice in the last 7 weeks of feeding (*p* < 0.05; Figure 5A). By the end of the 10-week feeding of the HFD, significant reductions in body weight gain and fat mass, especially fat mass of BAT and EWAT, were observed in the APOA4-Tg mice in comparison with WT mice (*p* < 0.05; Figure 5B,C and Table 3).

**Figure 6:**
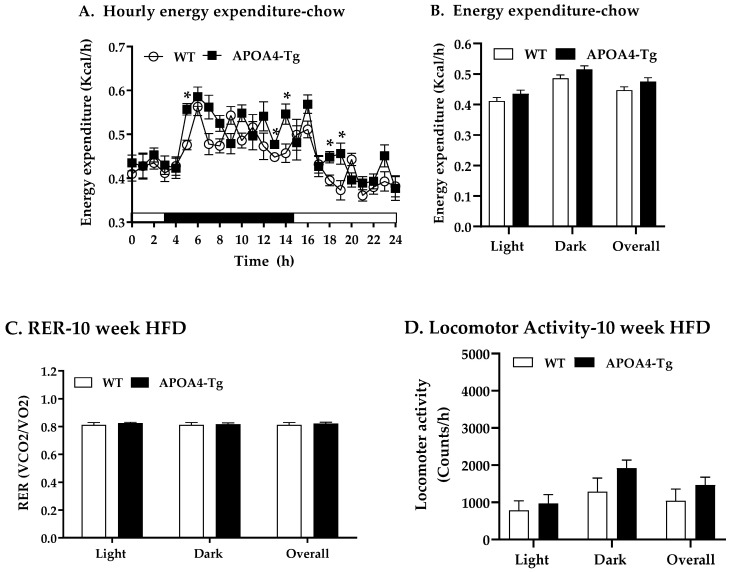
Hourly energy expenditure (**A**), average energy expenditure (**B**), average respiratory exchange ratio (RER, VCO2/VO2) (**C**), and locomotor activity (**D**) in mice during light and dark phases of the 10^th^ week of HFD feeding at 28 °C. Energy expenditure was covaried for lean body mass. Data are expressed as mean ± SEM. *n* = 4 or 5 per group. * Represent significant differences relative to WT mice (*p* < 0.05).

To examine if overexpression of APOA4 regulates energy expenditure in HFD-fed mice, energy expenditure, RER, and locomotor activity of WT and APOA4-Tg mice fed 10 weeks of an HFD were determined. In contrast to the percent lean mass normalized by body weight (Figure 5C), no significant difference in lean body mass not normalized by body weight between APOA4-Tg mice (21.3 ± 1.0 g) and WT mice (20.3 ± 0.8 g) was observed. Relative to the WT mice, APOA4-Tg mice fed an HFD exhibited increased energy expenditure at several time points during HFD feeding (*p* < 0.05, Figure 6A), but no significant difference in average of EE in light or dark phases or overall were observed between WT and APOA4-Tg mice (Figure 6B). Additionally, RER and locomotor activity in these APOA4-Tg mice were comparable to their controls (Figure 6C,D). In addition, plasma levels of TG and leptin in APOA4-Tg mice were lower than in WT mice (*p* < 0.05; Table 3). Daily caloric intake and plasma cholesterol levels were similar between the APOA4-Tg and WT mice (Table 3). These findings suggest that chronic consumption of an HFD induces plasma APOA4 levels in APOA4-Tg mice and the action of APOA4 stimulates norepinephrine levels and AMPKα-dependent pathway, elevates intracellular lipolysis, UCP1-dependent BAT thermogenesis and energy expenditure at several time points, attenuates an HFD-induced rise in body weight gain, fat mass and plasma lipids, and are independent of caloric intake after 10 weeks of HFD feeding.

## 3. Discussion

Although APOA4 is a well-known satiating protein that enhances thermogenesis and fatty acid uptake in BAT of chow-fed mice [31,38], the role of APOA4 in the regulation of BAT thermogenesis and lipid metabolism in HFD-fed obese mice remained elusive, in part because APOA4 levels are normally suppressed by chronic feeding of an HFD [34,35,36]. The present experiments tested the hypothesis that non-suppressible expression of mouse APOA4 in the small intestine, with a subsequent rise in plasma APOA4, would increase BAT thermogenesis and consequently reduce body weight and plasma lipids in HFD-fed mice. This series of experiments demonstrated that male APOA4-Tg mice maintained increased levels of plasma APOA4, correlating with increased UCP1-dependent BAT thermogenesis (reflected as an increase in BAT UCP1), in particular when fed an HFD, as well as a decrease in plasma TGs, which over the course of 10 weeks also resulted in reduced plasma lipid levels, body weight, and fat mass in comparison with their WT control group on the same diet, all without altering caloric intake. Since BAT function is sex-dependent [39], the effect of APOA4 in female APOA4-Tg mice remains to be tested.

In response to dietary challenge, UCP1 expression in BAT is increased in mice [40]. HFD induces BAT UCP1 levels [40]. Our previous reports demonstrated that APOA4-KO mice exhibited reduced levels of UCP1-dependent BAT thermogenesis in response to acute feeding of dietary lipids or one week of HFD feeding [32,41], demonstrating that peripheral and/or central APOA4 may elevate UCP1-dependent BAT thermogenesis. In the current experiments, APOA4-Tg mice had comparable UCP1-dependent BAT thermogenesis, energy expenditure, and food intake relative to their WT controls when they were maintained on chow diets at 21 °C. When fed an HFD for 10 weeks and housed at 21 °C, no significant differences in weekly body weight, body weight gain, and daily food intake were observed between APOA4-Tg and WT mice (Supplementary Figure S1). Mice have minimal BAT activity at thermoneutrality, the range of ambient temperatures without regulatory changes in metabolic heat production or heat loss [15]. Because the BAT activity of chow- or HFD-fed mice housed at 21 °C was activated to produce extra heat for defending their body temperature, the possibility existed that the lack of difference in body weight gain between APOA4-Tg and WT mice fed 10 weeks of HFD was due to extra heat production for defending their body temperature when housed at 21 °C. Thus, APOA4-Tg and WT mice were housed at thermoneutrality (28–30 °C) for minimized induction of BAT activity by ambient temperature in the HFD feeding experiments. Chow-fed APOA4-Tg mice had comparable body weight, fat mass, and caloric intake relative to their WT mice when they were housed at 28 °C (our unpublished data).

APOA4 KO mice have impaired lipid-induced norepinephrine synthesis and UCP1-dependent BAT thermogenesis [32]. In contrast, acute injection of APOA4 stimulates sympathetic activity, norepinephrine synthesis, intracellular lipolysis, and BAT thermogenesis in BAT of chow-fed mice in response to dietary lipids [31]. The findings suggest that APOA4 may induce UCP1-dependent BAT thermogenesis through elevation of norepinephrine levels and intracellular lipolysis. In the current study, overexpression of APOA4 elevated UCP1-dependent BAT thermogenesis, as indicated by increased levels of BAT UCP1, but did not alter norepinephrine synthesis (as indicated by levels of TH), AMPKα pathway, intracellular lipolysis, body weight, and fat mass in mice after short-term intake of an HFD for 4 weeks. Although whether overexpression of APOA4 in the small intestine would result in the stimulation of sympathetic activity in BAT in HFD-fed mice remains unknown, the present study indicated that after chronic consumption of an HFD for 10 weeks, APOA4-Tg mice had increased norepinephrine synthesis, ATGL-induced intracellular lipolysis, and elevated UCP1-dependent BAT thermogenesis in HFD-fed mice. The findings suggest that overexpression of APOA4 may act on sympathetic nerves, leading to increased UCP1-dependent BAT thermogenesis through activation of an AMPKα1-dependent pathway for increased ATGL-dependent intracellular lipolysis.

APOA4-KO mice had attenuated energy expenditure and comparable locomotor activity after one-week or 20-week feeding of HFD at 21 °C [32,41], indicating that APOA4 may enhance energy expenditure but does not alter locomotor activity. The current experiment demonstrated that APOA4-Tg exhibited an increase in energy expenditure at several time points, but no alteration in the average of overall energy expenditure independent of locomotor activity when fed 10 weeks of an HFD at 30 °C. Although overexpression of APOA4 increased plasma APOA4 levels in the current experiment, and a central effect of APOA4 has been shown to elevate BAT thermogenesis [42], it is unlikely in the current experiments that APOA4-Tg mice exhibited enhanced BAT thermogenesis and energy expenditure induced by central APOA4, because APOA4 produced in the intestine and found in plasma of APOA4-Tg mice cannot cross the blood brain barrier [43]. Consistent with previous findings [27,32,38], the present study indicated that overexpression of APOA4 did not inhibit daily caloric intake in APOA4-Tg mice when they were maintained on a chow diet, 4 weeks of an HFD, or 10 weeks of an HFD. APOA4-KO mice had comparable RER in our previous report [32]. The current study demonstrated that APOA4-Tg mice had comparable RER when fed a chow diet or an HFD, suggesting that no significant differences in energy substrates are used for heat production. During 10 weeks of HFD feeding, APOA4-Tg mice exhibited reduced body weight starting at 4 weeks. In contrast, APOA4-Tg mice had comparable body weight to their WT controls during 4 weeks of HFD feeding. This difference may have been due to the difference in the ages of the mice at the start of the HFD feeding. Mice were 10 weeks of age at the start of HFD feeding in the 10-week study while mice were 16 weeks of age at the start of HFD feeding in the 4-week study. The findings suggest that the reduction of body weight gain in APOA4-Tg mice with elevation of energy expenditure at several time points is independent of caloric intake and locomotor activity after 10 weeks of HFD.

Enhancement of BAT thermogenesis reduces hypertriglyceridemia and protects against obesity-related atherosclerosis development [9,44,45,46,47]. TG in chylomicrons or very low-density lipoprotein (VLDL) are hydrolyzed by action of lipoprotein lipase (LPL), and free fatty acids are released to the circulation [48]. Sympathetic activation leads to upregulation of LPL activity [49,50]. APOA4 has been reported to activate BAT sympathetic activity and promote LPL-induced hydrolysis of triglyceride-rich lipoproteins [30,31,51]. BAT is the major fat depot for APOA4-induced fatty acid uptake through elevation of LPL expression [31]. APOA4-KO mice have normal fat absorption, VLDL production, and comparable levels of plasma triglyceride and leptin, but delayed chylomicron clearance, impaired proximal TG transport, and altered intestinal gene expressions related to TG transport when compared with their WT control group [32,41,52,53,54]. However, whether alterations in fat absorption, chylomicron clearance, TG transport, and intestinal gene expression would also be observed in APOA4-Tg mice remains unknown. The effect of overexpression of intestinal APOA4 in the attenuation of HFD-induced obesity through modulation of lipid transport and metabolism in the small intestine, adipose tissues, and liver needs to be investigated.

APOA4 overexpression did not significantly affect fasting levels of triglyceride and cholesterol in chow-fed mice at normal ambient temperature in a previous study [29]. In the current study, overexpression of endogenous APOA4 did not alter plasma cholesterol and leptin levels, though a reduction in plasma TG was observed when the APOA4-Tg mice were maintained on a chow diet at normal temperature. The difference in plasma TG levels in chow-fed APOA4-Tg mice between the current and previous study could be due to differences in genetic background resulting from more extensive C57BL/6J backcrossing, various environmental factors, or dietary lipid composition of the chow diets [29]. After feeding of atherogenic diet for 5 days, comparable plasma TG and cholesterol were found between these two genotypes [29]. When fed an atherogenic diet for 14 weeks, APOA4-Tg mice exhibited increased levels of plasma TG and cholesterol compared to WT males [29]. Thus, chronic feeding of the atherogenic diet may affect plasma lipids in APOA4-Tg mice. In the present experiment, after short-term intake of HFD for 4 weeks, overexpression of APOA4 attenuated plasma TG, possibly due to APOA4-induced fatty acid uptake by BAT [31]. Furthermore, plasma leptin and cholesterol in the APOA4-Tg mice were not altered after short-term intake of HFD. The current experiment demonstrated that chronic consumption of HFD increased plasma lipids and leptin in WT mice, consistent with the observations in previous reports [55,56]. In contrast, APOA4-Tg mice exhibited attenuation of an HFD-induced rise in the plasma lipids and leptin. It is possible that APOA4-Tg mice have reduced plasma TG resulting from elevated LPL activity in BAT which promote triglyceride clearance and fatty acid uptake by BAT. A more extensive, comparative analysis of chow- and HFD-fed mice at room temperature and thermoneutrality is needed to provide better insight into the lipid profiles of APOA4-Tg mice, while investigation of the effect of endogenous APOA4 in the downregulation of plasma lipids through upregulation of fatty acid uptake and LPL activity in BAT of APOA4-Tg mice is also required.

A previous report has shown that APOA1 also activates BAT thermogenesis and consequently reduces body weight gain and fat mass in HFD-induced obese mice [57]. In the present study, because chronic consumption of HFD for 10 weeks increased levels of plasma APOA4 and also APOA1 in APOA4-Tg mice, APOA4-Tg mice likely experience a mixed effect of APOA4 and APOA1 in elevation of BAT thermogenesis and downregulation of body weight gain and fat mass after 10 weeks of HFD feeding. When chylomicrons enter the circulation, approximately 25% of the APOA4 is transferred to high-density lipoprotein (HDL) [58]. APOA1 is the most abundant apolipoprotein in an HDL [59]. APOA4 and APOA1 promote reverse cholesterol transport from extrahepatic cells and tissues to the liver and intestine for excretion [25,58,59].In the APOA4-Tg mice after 10 weeks of HFD feeding, overexpression of APOA4 elevated levels of plasma APOA4 and APOA1 and consequently downregulated levels of plasma cholesterol, possibly due to the mixed effect of APOA4 and APOA1 in facilitating reverse cholesterol transport. Further investigation into the elevation of reverse cholesterol transport in APOA4-Tg mice is required.

Activating BAT thermogenesis in rodents and humans has a great potential to combat obesity and cardiovascular diseases [7,9,10]. In view of the observations made using APOA4-KO mice previously [32] and using APOA4-Tg mice in the current study, lipid-induced endogenous APOA4 may increase UCP1-dependent BAT thermogenesis and energy expenditure at several time points and reduce body weight gain, fat mass and plasma lipids. Collectively, the present findings suggest that increased levels of endogenous APOA4 can elevate UCP1-dependent BAT thermogenesis and consequently attenuate HFD-induced rises in body weight gain, fat mass and plasma lipids in obese mice independent of caloric intake.

## 4. Materials and Methods

### 4.1. Animals

Male APOA4-Tg mice and WT mice (C57BL/6J background) were generated in an AAALAC-accredited facility under conditions of controlled illumination (12:12 h light-dark cycle, lights on from 0600 to 1800 h). APOA4-Tg mice were kindly provided by Dr. Karen Reue at the University of California, Los Angeles, CA, USA [29] and were back-crossed for >10 generations onto a C57BL/6J genetic background. All mice were genotyped by polymerase chain reaction (PCR) analysis of tail deoxyribonucleic acid (DNA) [29]. All animals at ages between 10 and 20 weeks received free access to water and either a standard chow diet (14% fat by weight, 60% carbohydrates by weight, and 25% protein by weight; # Prolab RMH3000 5P00, LabDiet, St. Louis, MO, USA) or an HFD (60% lard fat by weight, 20% carbohydrates by weight, and 20% protein by weight; # D12492, Research Diets, Inc., New Brunswick, NJ, USA) for 4 or 10 weeks. Body weight was monitored with a top-loading balance (± 0.01 g, Adenturer SL, Ohaus Corp, Pine Brook, NJ, USA). All animal protocols were approved by the Institutional Animal Care and Use Committee at Ohio University and University of Cincinnati.

### 4.2. Body Weight, Fat Mass, Plasma Parameters, and BAT Thermogenic and Lipolytic Proteins in APOA4-Tg Mice Fed a Chow Diet

WT and APOA4-Tg mice (*n* = 7–8 per group) at the age of 15 weeks had free access to a standard chow diet and water for 4 weeks while housed at 21 °C. Body weight and caloric intake were recorded weekly. At the end of the experiment, body composition was measured by LF-50 body composition analyzer (Bruker, Billerica, MA, USA) and body weight was recorded. Food was removed for 5 h before plasma, interscapular BAT, EWAT, and IWAT were collected and stored at −80 °C for further determinations.

### 4.3. Body Weight, Fat Mass, Plasma Parameters and BAT Thermogenic and Lipolytic Proteins in APOA4-Tg Mice Fed an HFD for 4 Weeks

Body weight and caloric intake in WT and APOA4-Tg mice (*n* = 6–8 per group) at the age of 16 weeks were recorded weekly while maintained on an HFD for 4 weeks at 28 °C. At the end of the experiment, body composition was measured by LF-50 body composition analyzer. Food was removed for 5 h before plasma, interscapular BAT, EWAT, and IWAT were collected and stored at −80 °C for further determinations.

### 4.4. Body Weight, Fat Mass, Plasma Parameters, and BAT Thermogenic and Lipolytic Proteins in APOA4-Tg Mice Fed an HFD for 10 Weeks

WT and APOA4-Tg mice (*n* = 9–10 per group) at the age of 10 weeks received 10 weeks of HFD feeding while housed at 28 °C. At the end of the experiment, body composition was measured by LF-50 body composition analyzer. Food was removed for 5 h before plasma, interscapular BAT, EWAT, and IWAT were collected and stored at −80 °C for further determinations.

### 4.5. Thermogenic Protein and Plasma Apolipoprotein Determination

BAT protein was extracted with RIPA lysis buffer system (Santa Cruz Biotechnology, Dallas, TX, USA). Plasma and extracted BAT protein mixed with 4x SDS sample buffer were boiled for 10 min according to our published protocol [31]. For the determination of UCP1 and beta-tubulin proteins, extracted BAT proteins (200 µg) were incubated with 2 µL of UCP1 (Abcam^®^, Waltham, MA, USA) or beta-tubulin antibody (Invitrogen^®^, Waltham, MA, USA) using a Dynabeads Protein A immunoprecipitation kit (Invitrogen, Vilnius, Lithuania) overnight at 4 °C. The eluted sample (30µL) was loaded onto a 4–20% Tris-HCl gradient gel (Bio-Rad Laboratories, Hercules, CA, USA). For the measurement of APOA4 and APOA1 proteins, plasma (2 µL) was loaded onto a 4–20% Tris-HCl gradient gel. The SDS gels were run at 60 voltages for 1 h and at 100 voltages until the protein standards were well separated. Proteins were then transferred to a polyvinylidene difluoride membrane (Bio-Rad Laboratories, CA, USA) for 2 h at a constant current of 350 mA. After the membrane was incubated in a 5% blotting-grade blocker solution (Bio-Rad Laboratories, CA, USA), the membranes were then incubated with one of either rabbit or mouse polyclonal antibodies in 5% bovine serum albumin (BSA): beta-tubulin (1:1000 dilution, #2128L, Cell Signaling Technology, Inc., Denvers, MA, USA), UCP1 (1:1000 dilution, #50-173-4107, Proteintech, Rosemont, IL, USA), TH (1: 1000 dilution, #2792S, Cell Signaling Technology, Inc.), ATGL (1:1000 dilution, #2439S, Cell Signaling Technology, Inc.), HSL (1:1000 dilution, #4107S, Cell Signaling Technology, Inc.), polyclonal APOA4 (1:5000 dilution, #PA5-14554, Invitrogen, Waltham, MA, USA) and APOA1 antibodies (1:5000 dilution, #PA5-29557, Invitrogen). After incubation with the primary antibody overnight at 4 °C, the immunoblots were washed and then incubated with horseradish peroxidase-conjugated goat anti-rabbit antibody (1:5000 dilution, Dako Cytomation, Santa Clara, CA, USA) for 1 h at 25 °C. Detection was achieved using the enhanced chemiluminescence system (Immobilon western chemiluminescent HRP substrate, EMD Millipore Corporation, Billerica, MA, USA). A C-Digit blot scanner (Li-Cor Biosciences, Lincoln, NE, USA) was used for development and visualization of the proteins, and infrared imaging was used for protein quantification. For BAT immunoblots, UCP1 protein concentrations were normalized to beta-tubulin. For plasma apolipoproteins, the average of band intensity of apolipoproteins in WT mice was considered as 100%.

### 4.6. Plasma Parameters

Plasma leptin level was determined using a commercial mouse leptin ELISA kit (Crystal Chem, Elk Grove Village, IL, USA). Briefly, plasma (10 µL) was added to each well of a microtiter plate pre-coated with anti-leptin monoclonal antibodies, and the detection antibody was added. After incubation, absorbance was measured with a microplate reader (Synergy HT, BioTek Instruments, Inc., Richmond, VA, USA), and the final concentrations were calculated using standards provided with the ELISA kit. Triglyceride and cholesterol in the plasma were determined using Infinity triglyceride and cholesterol kits (Thermo Scientific, Middletown, VA, USA).

### 4.7. Energy Expenditure, RER, and Food Intake of Mice Fed a Chow or HFD for 10 Weeks

Energy expenditure, RER, locomotor activity, and food intake were determined in APOA4-Tg and WT mice at an age of 19 weeks fed a powdered chow (*n* = 8 per group), or during the 10th week of HFD feeding (*n* = 4 per group). These animals were acclimatized to individual metabolic cages of a comprehensive lab animal monitoring system (CLAMS, Columbus Instrument, Columbus, OH, USA) for 3 days. Energy expenditure, RER, locomotor activity, and food intake were then recorded at 16 min intervals for 2 days.

### 4.8. Quantitative RT-PCR

Total RNA from BAT of 5 h fasted mice was isolated using a RNeasy lipid tissue mini kit (Qiagen, Hidden, Germany) according to the manufacture’s protocol, and first-strand complementary DNA (cDNA) was synthesized from 1 µg total RNA using a iScript cDNA synthesis kit (Bio-Rad Laboratories) [60]. The sequences of the primers (Integrated DNA Technologies, Coralville, IA, USA) were according to our published protocols [32,42]. Quantitative PCR (qPCR) was performed in a 25 µL final reaction volume, including 4µL of 10-fold diluted sample cDNA, 1µL of 10µM forwarded or reverse primers, and iTaq universal SYBR green supermix (Bio-Rad Laboratories), using a Bio-Rad RT-PCR instrument. PCR conditions were conducted as follows: 95 °C for 10 min for one cycle, followed by 40 cycles of 95 °C for 15 s and 60 °C for 60 s. Threshold cycle readings for each of the unknown samples were obtained, and the results were analyzed in Excel using the ∆∆Ct method [35]. Levels of 36B4 mRNA from each sample were similar among all groups and were used as internal controls to normalize the mRNA levels.

### 4.9. Statistical Analysis

All values are presented as mean ± SEM. For body weight measurements, repeated measures analyses of variance (ANOVA), followed by a Sidak’s multiple comparisons test, were performed using GraphPad™ Prism (version 9.0, San Diego, CA, USA). For energy expenditure measurements, analysis of covariance (ANCOVA) was performed with lean mass as the covariate using SPSS version 28.0 software (SPSS Inc., Chicago, IL, USA) [61,62]. For end-point measurements, unpaired t-tests were performed using GraphPad™ Prism (version 9.0, San Diego, CA, USA). Differences were considered significant if the *p* value was <0.05.

## 5. Conclusions

Maintenance of mouse APOA4 levels in the small intestine and plasma elevates UCP1-dependent BAT thermogenesis and energy expenditure and attenuates HFD-induced gains in body weight, fat mass, and plasma lipids, leading to protection against HFD-induced obesity in mice.

## Figures and Tables

**Table 1:** Body weight, fat mass, and levels of plasma parameters in chow-fed mice.

Parameters	WT Mice	APOA4-Tg Mice
Body weight (g)	26.4 ± 0.37	26.1 ± 0.55
Daily caloric intake (Kcal)	12.7 ± 2.63	12.5 ± 3.60
Fat mass/body weight (%)	10.6 ± 0.77	11.3 ± 0.63
Lean mass/body weight (%)	77.1 ± 1.65	76.4 ± 0.69
BAT (g)	0.15 ± 0.01	0.12 ± 0.02
EWAT (g)	0.34 ± 0.01	0.29 ± 0.03
IWAT (g)	0.21 ± 0.01	0.19 ± 0.02
Leptin (ng/mL)	1.85 ± 0.60	1.93 ± 0.76
Triglycerides (mg/dl)	50.0 ± 4.01	29.9 ± 10.25 *
Cholesterol (mg/dl)	80.8 ± 5.05	74.2 ± 3.21

Body weight, caloric intake, and lean/fat mass were monitored when mice (*n* = 7–8 per group) were maintained on a chow diet at 21 °C. Body weight, tissues, and plasma were collected after a 5 h fast. Daily caloric intake is the average of seven days of caloric intake measured during the 4th week of chow diet feeding. Values are represented as mean ± SEM. Values with asterisks (*) represent significant differences relative to the WT mice (*p* < 0.05).

**Table 2:** Body weight, fat mass, and levels of plasma parameters in mice fed an HFD for 4 weeks.

Parameters	WT Mice	APOA4-Tg Mice
Body weight (g)	32.5 ± 1.32	32.88 ± 0.88
Daily caloric intake (Kcal)	8.9 ± 0.50	9.9 ± 0.42
Fat mass/body weight (%)	21.3 ± 0.81	23.3 ± 1.41
Lean mass/body weight (%)	66.8 ± 0.52	64.6 ± 1.56
BAT (g)	0.20 ± 0.02	0.31 ± 0.03
EWAT (g)	1.25 ± 0.28	1.15 ± 0.21
IWAT (g)	0.80 ± 0.17	1.02 ± 0.12
Leptin (ng/mL)	14.6 ± 1.28	17.3 ± 2.85
Triglycerides (mg/dl)	60.6 ± 3.1	43.1 ± 6.51 *
Cholesterol (mg/dl)	143.8 ± 12.7	145.6 ± 11.0

Body weight, caloric intake, and lean/fat mass were monitored when mice (*n* = 6–7 per group) were fed an HFD for 4 weeks at 28 °C. Body weight, tissues, and plasma were collected after a 5 h fast. Daily caloric intake is the average of seven days of caloric intake measured during the 4th week of HFD feeding. Values are represented as mean ± SEM. Values with asterisks (*) represent significant differences relative to the WT mice (*p* < 0.05).

**Table 3:** Body weight, fat mass, and levels of plasma parameters in mice fed an HFD for 10 weeks.

Parameters	WT Mice	APOA4-Tg Mice
Body weight (g)	40.6 ± 1.88	32.4 ± 0.91 *
Daily caloric intake (Kcal)	11.6 ± 0.70	11.4 ± 0.78
BAT (g)	0.53 ± 0.07	0.32 ± 0.05 *
EWAT (g)	2.47 ± 0.28	1.33 ± 0.24 *
IWAT (g)	1.39 ± 0.18	1.14 ± 0.15
Leptin (ng/mL)	25.5 ± 1.77	16.7 ± 1.10 *
Triglycerides (mg/dl)	66.9 ± 8.71	45.5 ± 4.30 *
Cholesterol (mg/dl)	159.3 ± 16.60	138.6 ± 14.84

Body weight and caloric intake were monitored when mice (*n* = 9–10 per group) received an HFD for 10 weeks at 28 °C. Body weight, tissues, and plasma were collected after a 5 h fast. Daily caloric intake is the average of seven days of caloric intake measured during the 10th week of HFD feeding. Values are represented as mean ± SEM. Values with asterisks (*) represent significant differences relative to the WT mice (*p* < 0.05).

## Data Availability

The data that support the findings of this study are available on request from the corresponding author.

## Supplementary Materials

The following supporting information can be downloaded at: https://www.mdpi.com/article/10.3390/ijms24044231/s1.

## Abbreviations

AMPK	Adenosine monophosphate-activated protein kinase
APOA4	Apolipoprotein A4
APOA4-Tg	Transgenic mice with overexpression of mouse APOA4 in the small intestine
APOA4-KO	Global knockout of APOA4
ATGL	Adipose triglyceride lipase
BAT	Brown adipose tissue
EWAT	Epididymal white adipose tissue
HDL	High-density lipoprotein
HFD	High-fat diet
HSL	Hormone sensitive lipase
IWAT	Inguinal white adipose tissue
LPL	Lipoprotein lipase
TG	Triglycerides
TH	Tyrosine hydroxylase
UCP1	Uncoupling protein 1
UCP3	Uncoupling protein 3
VLDL	Very low-density lipoprotein
WAT	White adipose tissue
WT	Wildtype
