# Supplementary material for: Increased BAT Thermogenesis in Male Mouse Apolipoprotein A4 Transgenic Mice
Source: Int J Mol Sci. 2023 Feb 20;24(4):4231. doi: 10.3390/ijms24044231 (PMC9959433; doi:10.3390/ijms24044231)
Supplement: Supplementary file 1 [file ijms-24-04231-s001.zip › ijms-2139859-supplementary.pdf]

Supplementary

Body Weight and Food Intake in APOA4-Tg Mice Fed an HFD for 10 Weeks and Housed at Normal Ambient Temperature

To examine whether overexpression of APOA4 in the small intestine can regulate body weight and food intake in HFD-fed mice at normal ambient temperature, body weight and food intake in WT and APOA4-Tg mice were monitored when fed a HFD for 10 weeks at 21°C. After 10 weeks of HFD, APOA4-Tg mice have comparable body weight, body weight gain and daily food intake relative to their WT controls (Supplementary Figure S1A-C).

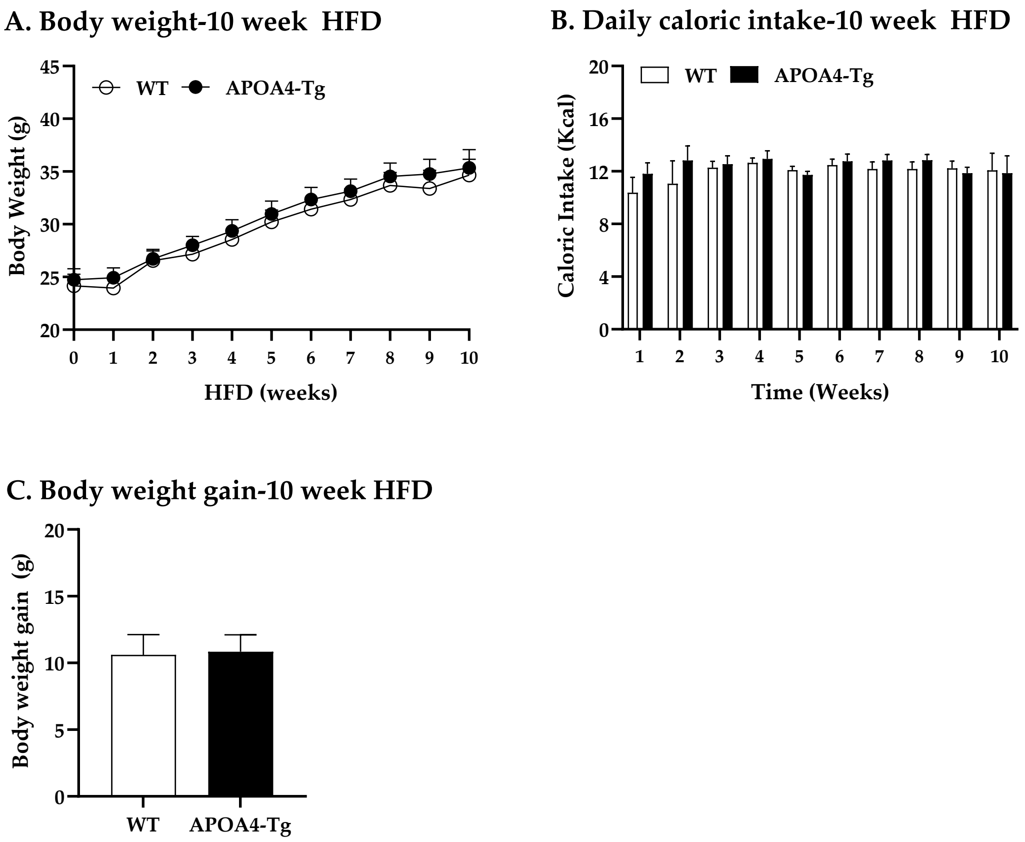

Supplementary Figure S1. Body weight (A) and daily caloric intake (B) in mice during a 10-week feeding of HFD at 21°C. Body weight gain (C) in mice fed an HFD for 10 weeks when mice were housed at 21°C. Data are expressed as mean ± SEM for 5-6 animals per group.
